# A novel risk score model based on four angiogenesis long non-coding RNAs for prognosis evaluation of pancreatic adenocarcinoma

**DOI:** 10.18632/aging.204387

**Published:** 2022-11-16

**Authors:** Guangbiao Cao, Yihang Chang, Guang Yang, Yong Jiang, Keqiang Han

**Affiliations:** 1 Department of Hepatobiliary Surgery, Songshan General Hospital, Chongqing, China

**Keywords:** pancreatic adenocarcinoma, long noncoding RNA, tumour angiogenesis, risk score model

## Abstract

Background: Long non-coding RNAs (lncRNAs) have been reported to play significant roles in tumour angiogenesis which prominently facilitates pancreatic adenocarcinoma (PAAD) progression.

Methods: The clinical PAAD data were obtained from TCGA database and clinical specimens of 122 PAAD patients. The Molecular Signatures Database v4.0 was used to identify angiogenesis-related long non-coding RNAs (ARLNRs). Survival-related ARLNRs (sARLNRs) were further validated by univariate and multivariate COX regression analyses. The expressions of CASC8, AC015660.1, Z97832.2 and PAN3-AS1 in PAAD cell lines and tissues were examined by qPCR. The correlations between sARLNRs (CASC8 and AC015660.1) and clinicopathological characteristics of the 122 PAAD patients were analyzed by the chi-square test and Fisher’s exact probability method.

Results: 590 lncRNAs were identified as ARLNRs, of which four sARLNRs were further used to establish an angiogenesis-related risk score model (ARRS), by which patients in the low-risk group have better survival probabilities than those in the high-risk group. The expression levels of CASC8 and AC015660.1 were significantly higher in PAAD cell lines and tumor tissues especially in patients with advanced grades and T-stages, while Z97832.2 and PAN3-AS1 were inverse. In addition, the higher expression of CASC8 and AC015660.1 prominently associated with the larger tumour size, and the more advanced grade and T-stage. However, the relevance between the sARLNRs (CASC8 and AC015660.1) expression and lymph node metastasis status was not significant.

Conclusions: In the study, we illuminate the clinical significance, angiogenesis relevance and prognosis-predictive value of four sARLNRs for PAAD. The results build a bridge between sARLNRs and tumour vascularization, and also establish a reliable and accurate risk scoring model for PAAD antiangiogenic strategy.

## INTRODUCTION

Tumour neovascularization, a complex pathological process in primary lesion or metastases, has been validated that plays prominent role on promoting cancer progression and evaluating tumour prognoses [1, 2]. New vessels not only provide tumour cells with abundant nutrition, but also form natural metastasis access to accelerate progression [3, 4]. Hence, angiogenesis-related precise treatment is promising for cancer comprehensive strategy. In addition, discovering more promising genetic biomarkers and targets is also of vital importance for pushing forward angiogenesis-related therapy.

Pancreatic adenocarcinoma (PAAD), as the most aggressive gastrointestinal carcinoma, is one of the most fatal of common malignancies [5, 6]. Although tremendous advance has been achieved in the mechanistic investigation of PAAD, early diagnosis and treatment are intractable yet, because of the heterogeneity and euangiotic intratumoral microenvironment [7, 8]. At present, expect for chemotherapy, there is no valid medical therapies for PAAD, besides, surgical intervention is merely appropriate for a small fraction of PAAD patients with resectable tumors [9]. Recently, a chain of promising angiogenesis-related biomarkers have been validated to play prominent roles on cancer early diagnosis and prognosis assessment. In consequence, a comprehensive knowledge of angiogenesis-related pathogenesis and the identification of novel angiogenetic markers and precise targets are promising for reaching better PAAD strategy.

Long non-coding RNAs (lncRNAs), which are a group of single-stranded nucleotide sequences exceeding 200 base pairs in length, participate in regulating a chain of biological processes, such as tumorigenesis, metastasis and angiogenesis. Emerging studies have highlighted the significance of lncRNAs in angiogenetic regulation [10–15]. Besides, certain angiogenesis-related lncRNAs (ARLNRs) are increasingly applied to prognostic assessment of malignancies [16]. LncRNA-FAM66C has been identified as a crucial regulator for reprogramming tumor microenvironment (TME) and hypoxia-related pathways in glioblastoma [17]. LncRNA MYLK-AS1 stimulating neovascularization by regulating miR-424-5p/E2F7 axis and activating VEGFR-2 signal transduction pathway in hepatocellular carcinoma [18]. LncRNA PAARH was validated to promote angiogenesis of hepatocellular carcinoma by inducing HOTTIP and activating HIF-1α/VEGF axis [19]. Besides, the prominent role of ferroptosis-related lncRNAs on predicting prognosis signature of PAAD has also been validated [20]. Therefore, ARLNRs as a category of potential biomarkers, are attaching increasing interest in the realm of angiogenesis-related targeted strategies.

## MATERIALS AND METHODS

### Human PAAD clinical samples and cell lines

PAAD patients’ tumor tissues and adjacent tissues were collected from 122 patients admitted to Songshan General Hospital between May 2018 and December 2021 (Table 1). The collected tissue samples were immediately frozen in liquid nitrogen until RNA extraction. HPDE6-C7 and PAAD cell lines (BXPC3, PANC1, ASPC1 and COLO357) were purchased from ATCC (Manassas, USA). DMEM, EMEM and 1640 basic medium supplemented with 10% fetal bovine serum (Gbico), 100 u/ml penicillin and 100 mg/ml streptomycin (Beyotime) was used to culture cell lines. Cells were incubated at 37° C in 5% CO_2_. The medium was changed every 3 days.

**Table 1:** The primer sequences of CASC8, AC015660.1, Z97832.2, PAN3-AS1 and β-actin.

**CASC8**	F primer (5’-3’)	CCAATCTAGGTTACCGGCAAG
	R primer (5’-3’)	TTCATGTGGCCTCTCATTGCT
**PAN3-AS1**	F primer (5’-3’)	CTGATGTTTGCGCTAATACCCT
	R primer (5’-3’)	TCTGCCGTTTGTGAACCTCTT
**AC015660.1**	F primer (5’-3’)	TTTCTCCCTGGCTGCTTCACA
	R primer (5’-3’)	GCATTCAGTCTGGAGTAGCCT
**Z97832.2**	F primer (5’-3’)	TCCTGAGATGAAGCTGGAAATCAA
	R primer (5’-3’)	AGTTTCTACGGTGGAGGGGT
**β-actin**	F primer (5’-3’)	AGGCCAACCGCGAGAAGATGACC
	R primer (5’-3’)	GAAGTCCAGGGCGACGTAGCAC

Note: F primer, forward primer; R primer, reverse primer.

### Transcriptome data download and preprocessing

Transcriptome RNA-sequencing data and clinical information of PAAD were downloaded from the TCGA database (https://portal.gdc.cancer.gov/), which contained 179 PAAD and 4 normal tissues, for subsequent analyses. RNA-seq results and clinical results were combined into a matrix file by a merge script in the Perl language (http://www.perl.org/).

### Angiogenesis-related long non-coding RNA extraction

The Molecular Signatures Database v4.0 was utilized to identify angiogenesis-related genes (ARGs). The correlation between ARGs and lncRNA levels was calculated by Pearson correlation analysis. A standard of |r|>0.3 and P<0.05 was used for ARLNR identification.

### Acquiring the survival-related ARLNRs (sARLNRs) and establishing the angiogenesis-related risk score model (ARRS)

ARLNRs with remarkable survival significance were served as sARLNRs in PAAD patients. sARLNRs were screened by univariate COX regression analysis (*P* < 0.05). sARLNR were further divided into protective and detrimental portions according to Hazard ratio (HR). In addition, sARLNRs are further screened by multivariate analysis, and the ARRS was established by sARLNRs regraded as independent prognostic indicators. Based on the different expressions of sARLNRs, we developed an ARRS to separate PAAD patients into high-risk group and low-risk group. The formula of ARRS construction was as followed, [level of AC015660.1 * (0.276515)] + [level of CASC8 * (0.373895)] + [level of PAN3-AS1* (-0.44547)] + [level of of Z97832.2 * (-0.61251)]. Patients were separated into high-risk group and low-risk group by the median risk score of ARRS.

### Real-time quantitative PCR

RT-qPCR was performed as previously described [21, 22]. Trizol (Invitrogen) was used to extract RNA from PAAD cell lines and tissues according to the manufacturer’s instruction. cDNA Synthesis Kit (TaKaRa) combined with 1μg RNA was utilized to reverse transcribed cDNA. The qPCR was performed on an ABI 7500 real-time PCR system (Applied Biosystems) according to the SYBR-Green method (TaKaRa). Relative expression levels of ARLNRs normalized to β-actin was calculated by the 2^−ΔCt^ method. The sequences were illustrated in Table 1.

### Bioinformatics analysis

OS of patients in the high-risk group and the low-risk group was assessed via Kaplan-Meier curve. ROC curve was utilized to estimate the sensitivity and specificity. Gene set enrichment analysis (GSEA) was used to explore the underlying pathways of ARRS. Univariate and multivariate Cox regression analyses and PCA were utilized for identifying independent prognostic factors of PAAD patients.

### Statistical analysis

Statistical analysis was conducted by SPSS21.0 software (SPSS 21.0) and GraphPad Prism8 (GraphPad prism 8, La). The difference comparison of two or more groups was performed by Student T-test, ANOVA and post-hoc test (Boferroni method). The correlations between sARLNRs and clinicopathological characteristics of PAAD patients were analyzed by the chi-square test and Fisher’s exact probability method. *P*<0.05 was considered a significantly statistical difference.

### Availability of data and materials

Authors can provide all of datasets analyzed during the study on reasonable request.

## RESULTS

### Seven sARLNRs are correlated to PAAD prognosis

**Figure 1:**
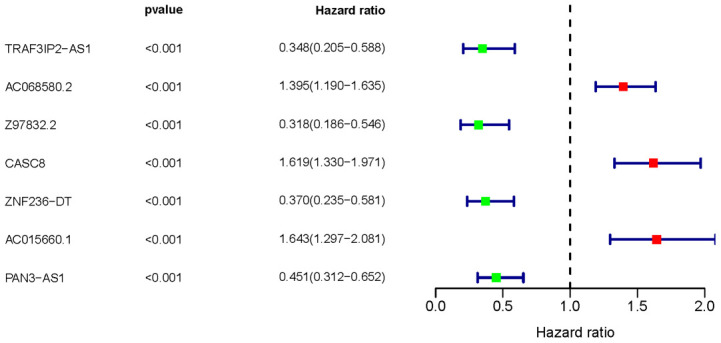
**Survival-related ARLNRs forest plot.** The hazard ratios of sARLNRs (TRAF3IP2-AS1, AC068580.2, Z97832.2, CASC8, ZNF326-DT, AC015660.1 and PAN3-AS1) were demonstrated in the forest plot. Red parts represent up-regulated sARLNRs, and green parts represent down-regulated sARLNRs.

Following analyzing PAAD transcriptome data of TCGA database, we identified 72 ARGs, of which 590 lncRNAs were further verified as ARLNRs via Pearson correlation analysis. Based on univariate COX Regression analysis, we then verified 7 ARLNRs that were correlated to prognoses of PAAD patients, such as TRAF3IP2-AS1, AC068580.2, Z97832.2, CASC8, ZNF326-DT, AC015660.1 and PAN3-AS1. The associations between these sARLNRs and prognoses are illustrated in the forest map clearly (Figure 1). AC068580.2, CASC8 ANS and AC015660.1 increase mortality risk of PAAD patients, while TRAF3IP2-AS1, Z97832.2, ZNF326-DT and PAN3-AS1 are positively correlated to OS.

### PAAD patients in the high-risk group show poor prognoses

**Figure 2:**
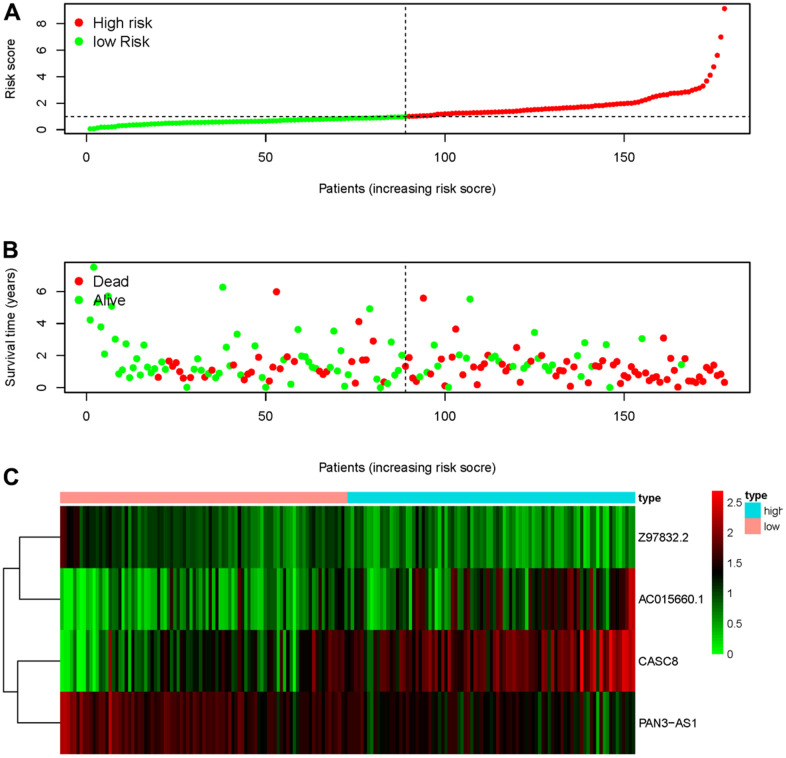
**ARRS was established based on sARLNRs.** The distribution of risk score in high-risk group and low-risk group (**A**). Survival status of the low-risk group and high-risk group (**B**). The heatmap of sARLNRs in ARRS (**C**).

**Figure 3:**
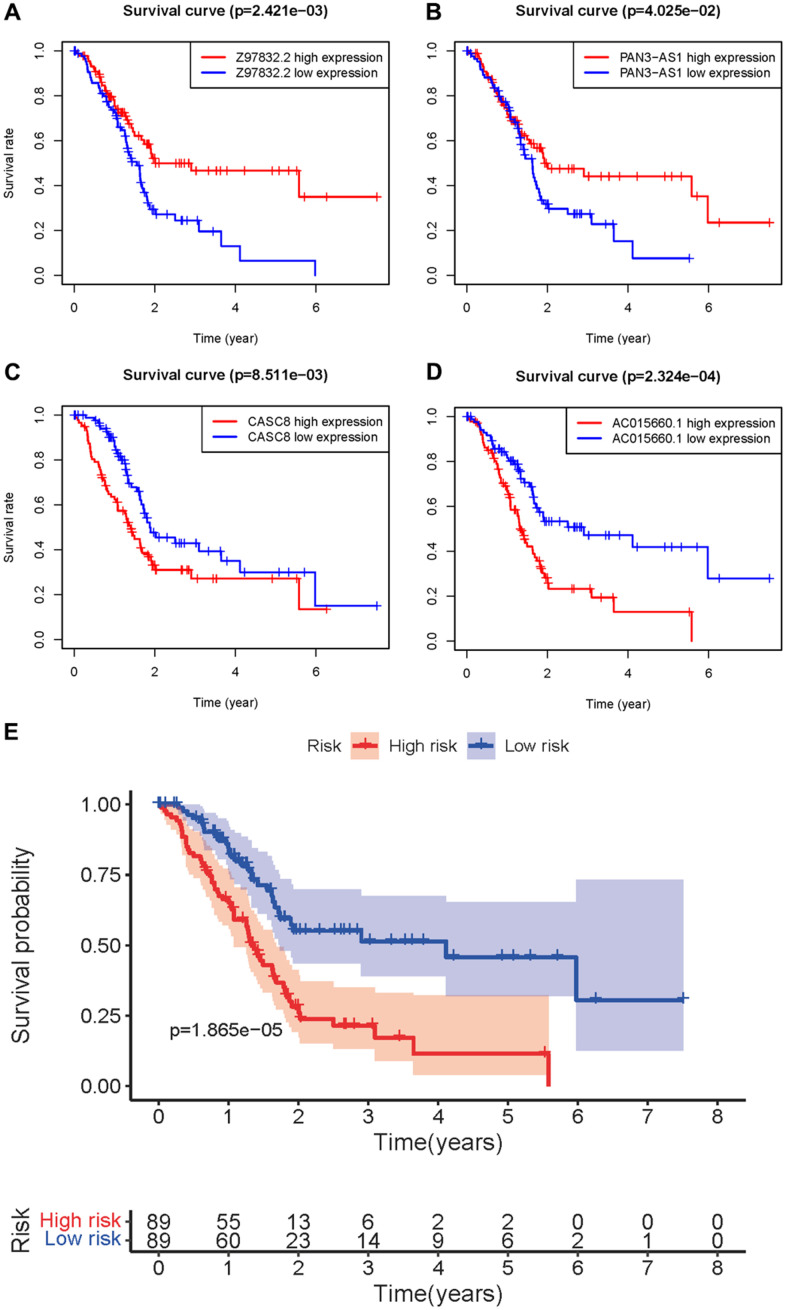
**Survival curve of sARLNRs and ARRS.** Kaplan-Meier survival curves of Z97832.2 (**A**), PAN3-AS1 (**B**), CASC8 (**C**) and AC015660.1 (**D**). The results showed the high expressions of Z97832.2 and PAN3-AS1 were correlated with a favorable prognosis, while the high expressions of CASC8 and AC015660.1 showed the opposite results. (**E**) Survival curve of the high-risk group and low-risk group. The results showed that the high-risk group of PAAD patients have a poor prognosis.

Four sARLNRs (Z97832.2, CASC8, PAN3-AS1 and AC015660.1) among the 7 sARLNRs were used to establish the ARRS, by which PAAD patients were separated into the high-risk group and the low-risk group (Figure 2A). In addition, the mortality rate of PAAD patients constantly decreased with lower risk score (Figure 2B). Along with the increasing risk score, the expression levels of AC015660.1, and CASC8 were enhanced, while Z97832.2 and PAN3-AS1 expressed decreasingly (Figure 2C). The low expression of Z97832.2 (Figure 3A) and PAN3-AS1 (Figure 3B) revealed the poor prognoses of PAAD patients, while the low expression of CASC8 (Figure 3C) and AC015660.1 (Figure 3D) showed opposite results. The survival curve of patient in the high-risk group was remarkably lower than patient in the low-risk group (Figure 3E). Therefore, the ARRS based on Z97832.2, CASC8, PAN3-AS1 and AC015660.1, to some extent, can accurately reflect prognoses of PAAD patients.

### ARRS is closely correlated to clinical features of PAAD

**Figure 4:**
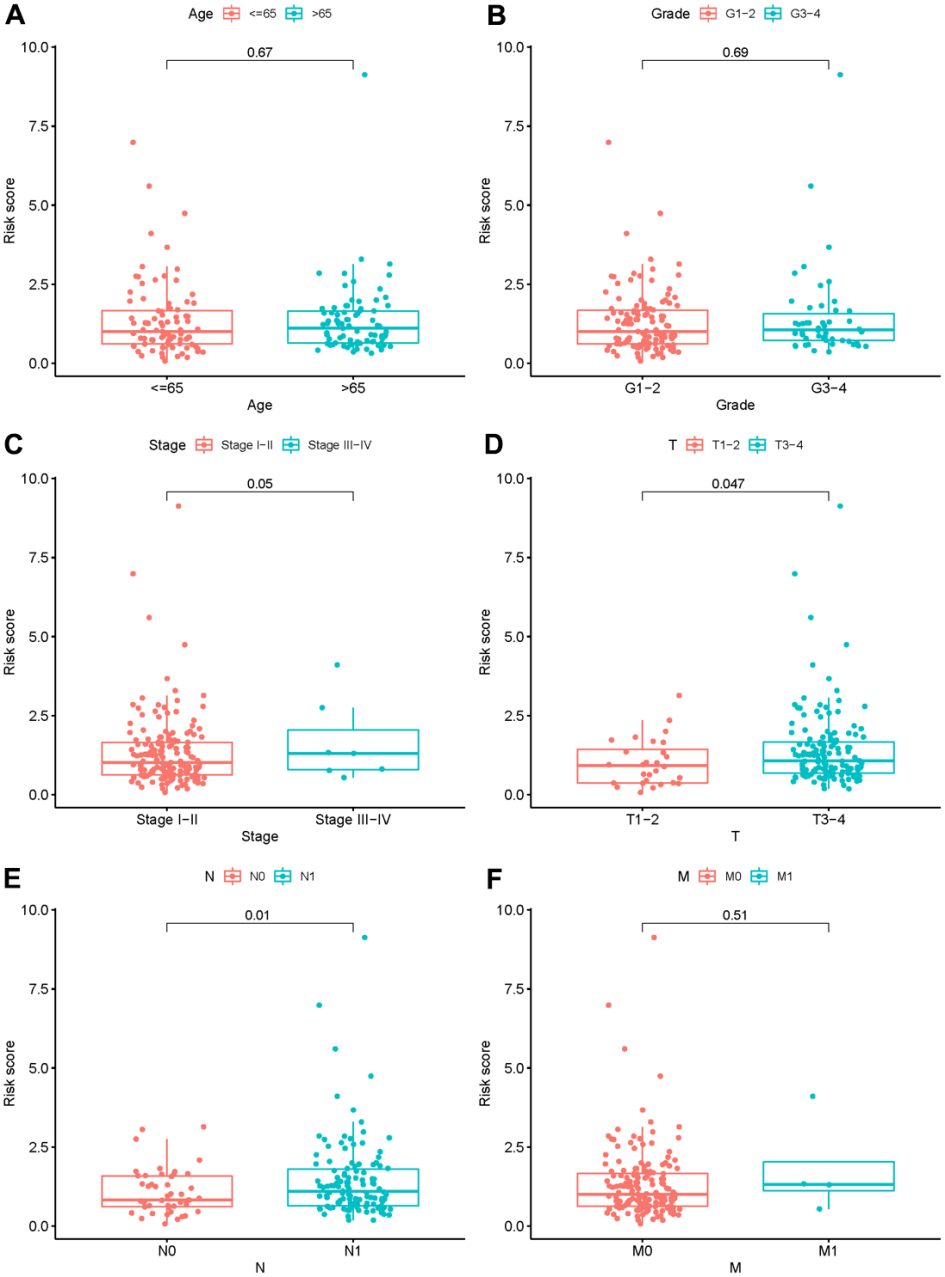
**The relationship between the risk score and clinical features.** Relationships between risk score and age (**A**), grade (**B**), stage (**C**), T- stage (**D**), N- stage (**E**) and M- stage (**F**).

**Figure 5:**
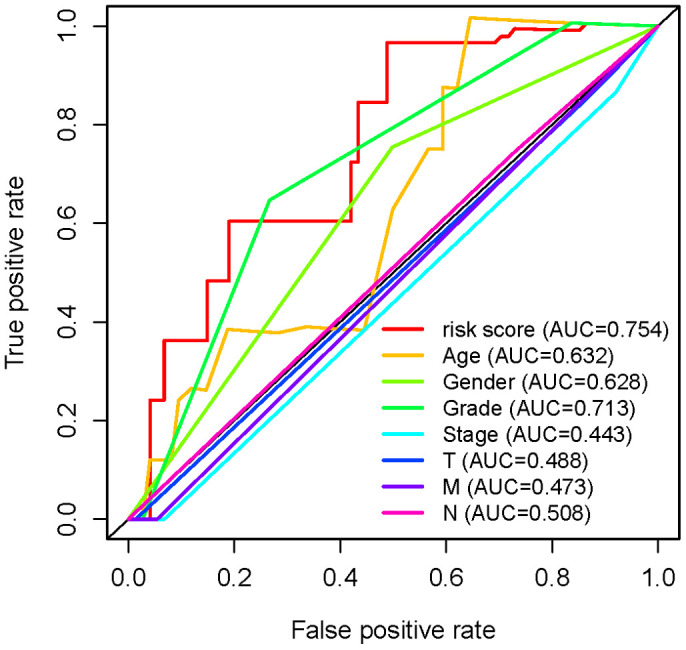
**Receiver operating characteristic (ROC) curve.** The AUC of risk score, age, gender, grade, stage, T-stage, M-stage and N-stage are 0.754, 0.632, 0.628, 0.713, 0.443, 0.488, 0.473 and 0.508 respectively.

**Figure 6:**
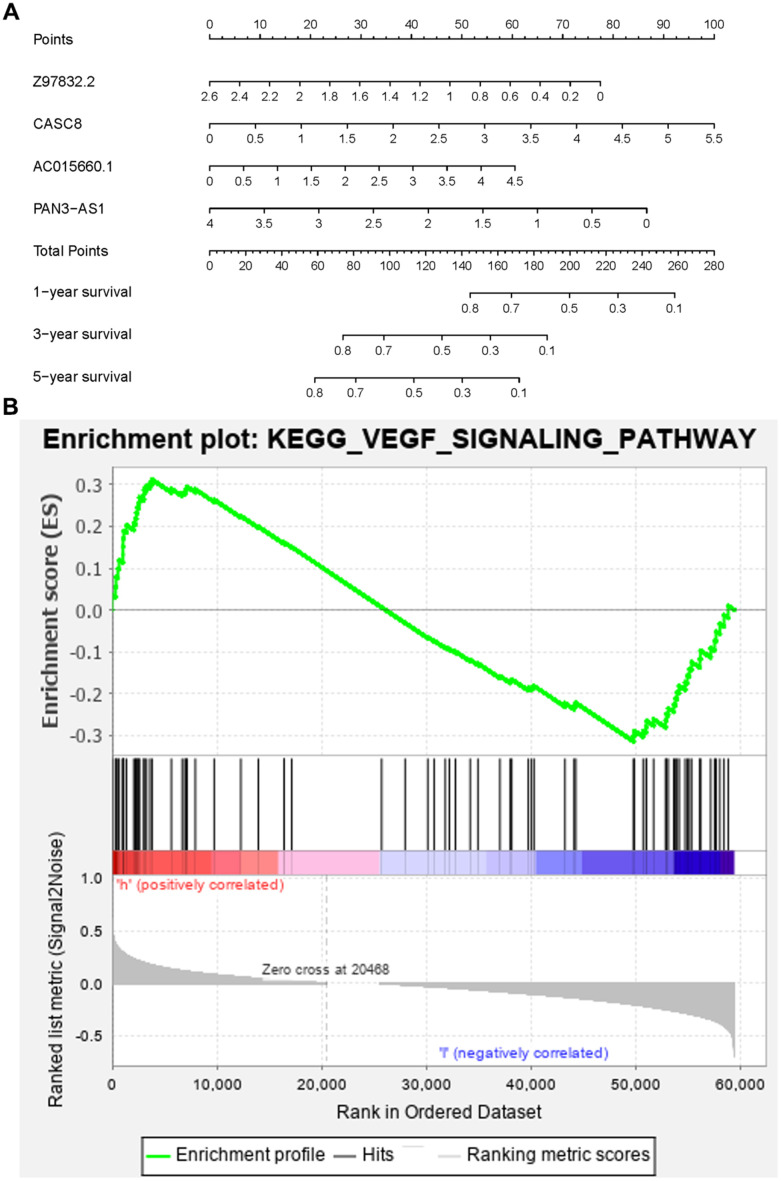
**The nomogram and KEGG pathway analysis.** The nomogram predicted the 1-,3- and 5- year survival rates of PAAD patients (**A**). The high-risk group had positive relations with the VEGF pathway (**B**).

To verify the clinical significance of ARRS, we detect the correlation of ARRS and clinical characteristics. We found that the higher risk score was correlated to the advanced stage, T-stage, N-stage and M-stage (Figure 4C–4F). However, there were no significant differences in grade and age (Figure 4A, 4B). Besides, the results of univariate and multivariate analysis illustrated that only risk score was remarkably associated with OS (Table 2). As the ROC curves showed, the AUC of risk score, age, gender, grade, stage, T-stage, M-stage and N-stage are 0.754, 0.632, 0.628, 0.713, 0.443, 0.488, 0.473 and 0.508 respectively, representing the accuracy of the ARRS (Figure 5). In addition, we normalized the points of ARRS ranging from 0 to 100, and calculated the 1-year, 3-year and 5-year survival probabilities by drawing expression of sARLNRs line between the total points axis and each prognosis axis (Figure 6A). The nomogram provided a novel diagnosis method at the genetic level for clinical doctors to estimate the prognoses of PAAD patients. In addition, we performed KEGG analysis to investigate mechanisms of the four sARLNRs included in the ARRS, and discovered that the high-risk group was associated with the activation of the VEGF signaling pathway (Figure 6B). Together with the above findings, we found that ARRS is not only associated with TNM stages of PAAD, but also displays the angiogenetic correlation.

**Table 2:** Univariate and multivariate COX analysis of PAAD patients.

	**Univariate analysis**				**Multivariate analysis**			
**Variables**	**HR**	**HR 95% low**	**HR 95% high**	**P value**	**HR**	**HR 95% low**	**HR 95% high**	**P value**
**Age**	1.018628	0.989299	1.048826	0.215632	1.027764	0.997404	1.059049	0.073443
**Gender**	1.223326	0.659408	2.269496	0.522628	1.374561	0.661768	2.855108	0.393650
**Grade**	1.445900	0.953290	2.193066	0.082759	1.216647	0.754200	1.962649	0.421544
**Stage**	0.955028	0.527062	1.730493	0.879406	0.222853	0.019778	2.511006	0.224406
**T-stage**	0.990137	0.414295	2.366358	0.982211	2.296308	0.295395	17.85077	0.426902
**M-stage**	1.061863	0.253749	4.443581	0.934496	17.41579	0.102794	2950.646	0.275195
**N-stage**	1.644979	0.785281	3.445847	0.187076	2.180775	0.812202	5.855412	0.121815
**Risk score**	1.709410	1.314281	2.223332	6.39e-05	1.802043	1.303285	2.491671	0.000367

Note: HR, Hazard Ratio.

### The expression levels of CASC8 and AC015660.1 are higher in tumour tissues and cell lines of PAAD

**Figure 7:**
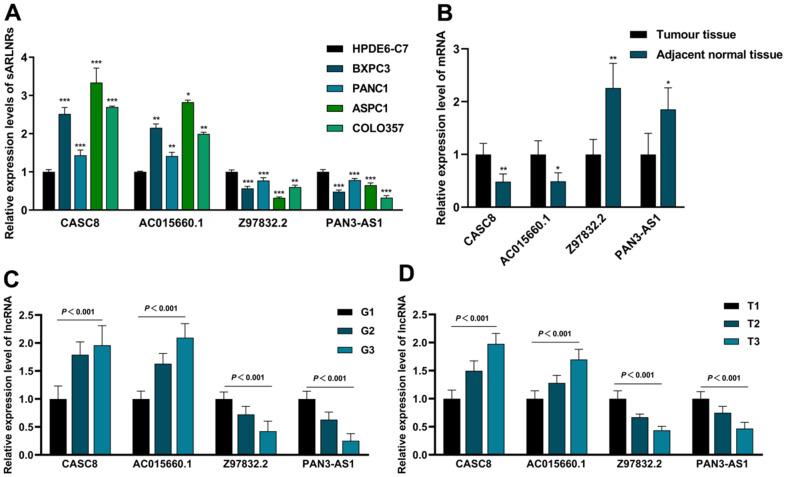
**The expression levels of CASC8, AC015660.1, Z97832.2 and PAN3-AS1 in cell lines and clinical samples.** The qPCR results of CASC8, AC015660.1, Z97832.2 and PAN3-AS1 in PAAD cell lines (BXPC3, PANC1, ASPC1 and COLO357), pancreatic epithelium cell (HPDE6-C7) (**A**), PAAD tumor tissues, tumor tissues with different grade and T-stage and adjacent normal tissues (**B**–**D**). CASC8 and AC015660.1 were highly expressed in PAAD cell lines (**A**), PAAD tissues (**B**), advanced grade (**C**) and advanced T-stage (**D**). While the expression of Z97832.2 and PAN3-AS1 increased in pancreatic epithelium cell (**A**), adjacent normal tissues (**B**), early grade (**C**) and early stage (**D**).

Next, we examined the levels of CASC8, AC015660.1, Z97832.2 and PAN3-AS1 in HPDE6-C7 cell line, various PAAD cell lines and tumour and adjacent tissues of PAAD. We found that CASC8 and AC015660.1 expressed significantly higher in BXPC3, PANC1, ASPC1 and COLO357 cell lines than those in HPDE6-C7, while the expression of Z97832.2 and PAN3-AS1 showed the opposite results (Figure 7A). Furthermore, consistent with the results in cell lines, we found that Z97832.2 and PAN3-AS1 expressed lowly in PAAD tumour tissues than those in adjacent normal tissues, but CASC8 and AC015660.1 showed the higher levels in tumour tissues (Figure 7B). Therefore, the expression differences of the four sARLNRs in cell lines and clinical specimens are accordant to the analyzing result based on database, further reflecting the reliability and accuracy of the ARRS.

### The expression levels of CASC8, AC015660.1, Z97832.2 and PAN3-AS1 are closely associated with tumour size, grade and T-stage

To further detect the clinical significances of the sARLNRs, we detected the expression levels of CASC8, AC015660.1, Z97832.2 and PAN3-AS1 in PAAD samples of various grades and T-stages. Compared with the adjacent normal tissues, CASC8 and AC015660.1 expressed higher in PAAD tumor tissues with more advanced grades (Figure 7C) and T-stages (Figure 7D), however, Z97832.2 and PAN3-AS1 dropped. Furthermore, we also analyzed the relevance to different clinicopathological characteristics. We detected that the higher expression of CASC8 and AC015660.1 prominently correlated to the larger tumour size, and the more advanced grades and T-stages (Table 3). Consistent with the results in database, we didn’t detect the prominent relevance between the sARLNRs and lymph node metastasis status. In general, the four sARLNRs-established ARRS is strongly related to clinicopathological features of PAAD, so as to relatively accurately reflect prognoses of PAAD patients.

**Table 3:** Relationship between CASC8 and AC015660.1 expression and clinicopathologic factors of PAAD patients.

**Parameter**	**N**	**Average expression of sARLNRs**		***P* value**
		**Low**	**High**	
**Gender**				0.260
**Male**	79	42	37	
**Female**	43	18	25	
**Age (year)**				0.720
**< 60**	46	21	25	
**≥ 60**	76	32	44	
**Tumor size (cm)**				**<0.001**
**≤4**	103	76	27	
**>4**	19	3	16	
**Grade**				**<0.001**
**1**	81	65	16	
**2**	36	14	22	
**3**	5	1	4	
**T-stage**				**<0.001**
**T1**	68	55	13	
**T2**	35	21	14	
**T3**	19	3	16	
**Lymph node status**				1.000
**Negative**	81	53	28	
**Positive**	41	27	14	

Note: The bold number represents the *P*-values with significant differences.

## DISCUSSION

Amount of evidence has unraveled that tumour-related angiogenesis prominently promoted cancer progression, including PAAD. A chain of angiogenic key regulating factors, such as vascular endothelial growth factor (VEGF), hypoxia-inducible factor 1 (HIF1) and fibroblast growth factor (FGF), have also been verified to closely associated with PAAD prognosis. Therefore, in the past decades, increasing numbers of antiangiogenic inhibitors targeting to these key regulators are constantly approved for clinical therapy, especially for those vessel-rich tumours [20, 23–26]. Belzutifan has been demonstrated to inhibit angiogenesis by attenuating the binding between Per-ARNT-Sim-B and HIF-2α [27].

Furthermore, tumour-related angiogenesis mechanisms are increasingly revealed, which also provide fundament for identifying more promising therapeutic targets. Marina recently found that suppression of endothelial cell focal adhesion kinase expression reduced PAAD liver metastasis by attenuating gemcitabine-mediated angiogenetic factors [28]. Chen et al. revealed angiogenetic mechanism in PAAD microenvironment, and they found that PAAD-secreted exosomes containing miRNA-30b-5p activate angiogenetic activities of endothelial cells via inhibiting the expression of gap junction protein GJA1 [29]. The study from Marjorie validated that targeting cancer-associated fibroblasts or inducing the endothelial-mesenchymal transition reversion process can attenuate angiogenesis of PAAD [30].

Recently, emerging studies have highlighted the directly and indirectly regulating effects of lncRNAs in angiogenesis process by targeting various angiogenetic molecules. LncRNA MYLK-AS1 was verified to promote hepatocellular carcinoma angiogenesis by targeting miR-424-5p/E2F7 axis and induce VEGFR-2 expression [18]. Moritz recently reviewed the potential of small extracellular vesicles containing lncRNAs as a series of biomarkers and proposed the possibility of small extracellular vesicle as delivery vehicles for lncRNA-based PAAD strategy [31]. Furthermore, a growing body of ARLNRs are potential for cancer assessment of diagnosis and prognosis. For example, AC005625.1 and AC008760.1 were significantly related to endothelial cells percentage, tumour size, muscle invasion status and poor prognosis in clear cell renal cell cancer in bladder urothelial carcinoma [16].

In the present study, we identify four sARLNRs with prominent clinical significance for PAAD. By the ARRSM established based on these sARLNRs, PAAD patients are separated into the high-risk group and the low-risk group, reflecting the distinct OS. Additionally, we verified the clinical significance of the four sARLNRs and found that the expression levels of CASC8 and AC015660.1 were significantly higher in PAAD cell lines and tumor tissues especially in patients with advanced grades and T-stages, while Z97832.2 and PAN3-AS1 were inverse. LncRNA-CASC8 polymorphisms have been demonstrated to increase the risk of esophageal cancer and lung adenocarcinoma [32, 33], but the role in PAAD is first revealed in the present study. AC015660.1 was identified as a novel inflammation-related lncRNAs to predict the prognosis of gastric carcinoma patients [34], furthermore, we demonstrate its potential, as an angiogenesis-related lncRNA, for assessing PAAD prognosis here. PAN3-AS1 has ever been verified as a ferroptosis-related lncRNA to predict the immune landscape in PAAD [20]. Here, we, for the first time, establish an ARRSM based on CASC8, AC015660.1, Z97832.2 and PAN3-AS1, which not only offer more promising targets, but also better assess tumour vascularization status and prognosis for PAAD patients.

Although our findings reveal the values of ARRSM for prognosis evaluation of PAAD patients and verified clinical significance and angiogenetic relevance of CASC8, AC015660.1, Z97832.2 and PAN3-AS1 in cell lines and clinical specimens, some limitations are still needed to be further improved in subsequent study. We found that these angiogenesis-related lncRNAs are significantly differentially expressed in tumour cell or tumour tissue, but their levels and functions in tumour endothelial cells remain unknown. Furthermore, the approaches and underlying mechanisms by which these lncRNAs regulate angiogenesis in tumour environment, such as small extracellular vesicle dependence, extracellular matrix degradation or others, are also needed to be further studied. Therefore, more *in vivo* and *in vitro* models should be constructed to elucidate mechanisms underlying angiogenesis in tumour microenvironment, besides, multiple omics assays are also needed to reveal the deeper and wider perspectives on tumour vascularization.

## CONCLUSIONS

In this study, we illuminate the promising roles of sARLNRs on prognosis evaluation for PAAD patients and determined the clinical significance and angiogenetic relevance, after the verification in 122 PAAD tissues and cell lines. The results build a bridge between sARLNRs and tumour vascularization, and also establish a reliable and accurate ARRSM for PAAD antiangiogenic strategy.
